# The Association of Thyroid Nodule with Non-Iodized Salt among Chinese Children

**DOI:** 10.1371/journal.pone.0102726

**Published:** 2014-07-28

**Authors:** Weimin Xu, Zexin Chen, Hui Liu, Liangliang Huo, Yangmei Huang, Xingyi Jin, Jin Deng, Sujuan Zhu, Wen Jin, Shanchun Zhang, Yunxian Yu

**Affiliations:** 1 Department of Endemic Diseases Control and Prevention, Hangzhou Center for Disease Control and Prevention, Hangzhou, Zhejiang, China; 2 Department of Epidemiology and Health Statistics, School of Public Health, School of Medicine, Zhejiang University, Hangzhou, Zhejiang, China; 3 Clinical Epidemiology and Biostatistics Center, The Second Affiliated Hospital, Zhejiang University, School of Medicine, Hangzhou, Zhejiang, China; Institute of Endocrinology and Metabolism, Iran, Islamic Republic Of Tehran

## Abstract

**Objective:**

The controversy that iodized salt may increase the risk of thyroid disorders has been aroused in China during the past decade. Most of studies focused on adult rather than children. We aimed to explore whether iodized salt was associated with an increased risk of thyroid nodule in Chinese children.

**Methods:**

The cross-sectional study was conducted in Hangzhou, China, in 2010. Iodized salt intake, urine iodine concentration (UIC) and thyroid nodule (by ultrasonography) were measured in 3026 children. The associations of iodized salt with thyroid nodule were evaluated using multiple logistic regression models.

**Results:**

The prevalence of thyroid nodule was 10.59% among Chinese children. Girls (11.89%) had higher prevalence of thyroid nodule than boys (9.26%). No significant association was observed between type of salt and thyroid nodule in pooled samples, boys and girls, respectively. Similar associations were observed between UIC and thyroid nodule. There was no significant association between milk consumption and thyroid nodule as well.

**Conclusion:**

The present study indicated that non-iodized salt may not increase the risk of thyroid nodules among Chinese children. Similar associations were observed between milk consumption, UIC and thyroid nodules.

## Introduction

Thyroid nodules are less common in children than those in adults, the prevalence of palpable thyroid nodules in childhood being about 1.5%, whereas it is 4–7% in adulthood [1], [2]. In contrast, thyroid nodules are more often malignant in childhood than in adulthood: 26% of thyroid nodules are malignant in children, while 5–10% in adults [2], [3]. Therefore, the early prevention of thyroid nodule in daily life of children is important and indispensable.

Several risk factors are associated with an increased risk of thyroid nodules; and among of them, iodine insufficiency is realized as an important one [3]. Iodine is an essential trace element required for normal activity of the thyroid gland. Iodization of salt is considered as the appropriate method for iodine fortification in prosperous countries [4]. The Universal Salt Iodization (USI) program was launched to prevent iodine deficiency disorders (IDD) in 1995, China. However, careful monitoring is essential because excessive iodine intake can also be disadvantageous and even a little deficiency in iodine intake have proven to be decisive for the prevalence of endemic goiter and other iodine-related thyroid diseases [5]–[7]. Similarly, experts of endocrinology in China have subjectively perceived that numbers of patients with thyroid nodule dramatically increased during the same period of USI, especially in coastal areas of China. Hence, the experts consensusally deemed that iodine intake was excessive among Chinese population. According to the guidelines of the World Health Organization (WHO), the United Nations Children's Fund, and the International Council for Control of Iodine Deficiency Disorders [8], iodine intake in part of Chinese residents were more than adequate (median urinary iodine excretion, 200 to 299 µg/L) or excessive (median urinary iodine excretion, >300 µg/L). But as Teng et al [9] reported, people with more than adequate and excessive iodine intake accounted on 0.2% and 0.5%, respectively. Consequently, a heated debate whether it is scientific and essential to keep launching the Universal Salt Iodization (USI) program throughout China was argued about [10]–[14]. Therefore, it is extremely important to explore whether USI was related to the high prevalence of thyroid nodule in China. Many previous studies conducted the relationship in adult population, and less of them focused on the children. Therefore, the aim of this study was to explore whether iodized salt was associated with an increased risk of thyroid nodule in Chinese children. Additionally, previous studies found that milk influenced urinary iodine concentrations significantly, suggested that milk might partly improve iodine intake in Spain [15], [16]. Thus, we also evaluate the effects of milk consumption on the prevalence of thyroid nodule. Further, a national survey in China reported that pubic hair development (PH 2) of boys was 11.24 years [17]; and the median ages of onset of Tanner stages 2 and 3 for pubic hair development were 11.16 (95% CI: 11.03–11.29) years and 12.40 (95% CI: 12.25–12.55) years, respectively [18]. Hence, in order to evaluate the effect of puberty, we did sub-analyses in two age groups (group 1: 6≤Year≤11; group 2: 12≤Year≤17).

## Subjects and Methods

### Population features

This study was conducted in Hangzhou, Zhejiang Province, China, which is located in the eastern of China. This city covers eight districts and five counties. There are 8.7 millions residents in Hangzhou city; 73.25% of them living in urban areas, 26.75% in rural areas. This study was designed to be representative of the civilian from Hangzhou and collected information on demographic characteristics, health status, iodine intake and thyroid disease. From March to October 2010, a total of 12,438 eligible individuals were recruited from greater Hangzhou, 9412 adults and 3026 children [19]. In present manuscript, we evaluated the association between type of salt and thyroid nodule among children (3026 adolescents).

### Subjects and study design

The study participants were recruited based on following strategy: There are eight districts and five counties in Hangzhou city. First, three sub-districts or towns were selected randomly from each district or county (including 12 districts or counties, except Binjiang district), respectively. Therefore a total of 36 sub-districts or towns were selected initially. Secondly, one community or village was randomly selected from each sub-district or town. Thirdly, 100 households from each community or village were randomly selected. Finally, 3600 households were recruited for interview. The family members of household were chosen based on the following criteria: (1) age ≥6 years old; (2) living for above 5 years at present residence. The exclusion criteria: (1) resident with coronary angiography (CAG) or endoscopic retrograde cholangiopancreatography (ERCP) in 6 months; (2) resident taking amiodarone drug; (3) resident with abnormal kidney function or serious illness. However, participants aged <18 years old were included in present manuscript.

The eligible family members of selected households were convened to administration center of village or community. The researchers introduced the study protocol and obtained written informed consent form from each participant. Meanwhile, the interview schedule was appointed with participants. The study protocol was approved by Institutional Review Board of Hangzhou Center of Disease Control and Prevention. This survey was carried out by well-trained personnel (including community clinic physicians, nurses, and public health doctors).

### Collection of epidemiological data and specimens

The participants were invited to local community health center before 8:00 am after 8-hour fasting overnight. Firstly, each participant provided 10 ml spot urine samples. Then, 5 ml venous blood was collected using vacuum blood collection tube. Secondly, the participants were interviewed for a structured questionnaire. The questionnaire covered the information about demographic characteristics and health status, including sex, age, nationality, physical activity, lifestyle, dietary habit, and personal or family history of thyroid disease (including time of diagnosis). Especially, information about type of salt, salt appetite and milk consumption was defined as following: the item–“Currently, which type of salt is consumed in your family?” was used for collecting information about type of salt. Three options for this item were provided: (1) only iodized salt; (2) non-iodized salt; (3) both of them. The question–“How about your appetite?” was used to evaluate salty appetite. Three options were provided: (1) salty; (2) moderate; (3) light.

Consuming milk ≤1 time per week was defined as having no habit of milk consumption, consuming milk ≥2 times per week was defined as having habit of milk consumption [19]. Eligible participants were interviewed face-to-face by well-trained investigators from CDCs.

### Measurements for iodine concentrations

For each household, table salt samples of at least 50 g were collected, which were then sealed and stored at room temperature away and kept in dark till to measurements. The concentration of iodine was measured by the colorimetric titration method. The collected urine sample was transferred in tightly capped plastic tubes barcode-labeled with a unique identifying number. Samples were stored in a refrigerator before transferring to the laboratory, where they were stored at −18°C until use. Urine iodine was measured using arsenic cerium catalytic spectrophotometry (WS/T107-2006) and expressed as micrograms of iodine per liter of urine. Epidemiologic criteria for assessing iodine nutrition based on median urinary iodine concentration are: iodine deficiency, <100 µg/L (Low); adequate iodine nutrition, 100–199 µg/L (Normal); above requirements, 200–299 µg/L (High); excess of iodine, ≥300 µg/L (Excess) [20].

Serum was obtained from vein blood after centrifugation and stored at −70°C until measurements. Serum thyroid stimulating hormone (TSH; normal range 0.35–5.50 mIU/L), free triiodothyronine (FT3; normal range 2.30–4.20 ng/L), triiodothyronine (T3; normal range 0.60–1.81 µg/L), free tetraiodothyronine (FT4; normal range 8.9–17.6 ng/L), tetraiodothyronine (T4; normal range 45.0–109.0 µg/L), thyroglobulin antibody (TgAb; normal range 0.0–60.0 KU/L), thyroid peroxidase antibody (TPOAb; normal range 0.0–60.0 KU/L) were only measured among participants from two district (Xiacheng and Jiangde), using the chemiluminescence immunoassay method, with electrochemical luminescence (Advia Centaur).

Anthropometric and physical examination data were obtained from each participant. Weight and standing height were measured in the standardized methods. Body mass index (BMI) was calculated as weight (kg)/height (m^2^). An ultrasound examination of the thyroid was performed to detect thyroid nodules, using a Sonoline Versa Pro (Siemens, Munich, Germany) with a 7.5-MHz, 70-mm linear transducer (effective length, 62 mm). The sonography was performed by one ultrasound doctor from county-level hospital in each district or county. All ultrasound doctors were trained before the investigation.

### Definition of variables

Diet pattern was classified as three groups: vegetarian, meat and moderate; vegetarian defined as subjects mainly consumed vegetable food; meat defined as subjects mainly consumed meat food; moderate defined as subjects consumed vegetable and meat food. In this study, the urine iodine concentration (median, interquartile) in subjects who consumed iodized salt, non-iodized salt, and both of them had 189.0(125.0, 275.9) µg/L, 115.0(81.0, 168.0) µg/L, and 149.0(103.0, 241.0) µg/L, respectively. Due to urine iodine concentration among subjects who consumed non-iodized salt and both of salt was very close, type of salt was grouped into two groups: iodized salt and non-iodized salt; non-iodized salt included subjects intermittently consumed iodized salt or consistently consumed non-iodized salt.

### Statistical analysis

The categorical and continuous variables were described as percentage and mean ± SD, respectively. Meanwhile, the categorical and continuous variables were compared between the subjects with and without thyroid nodules, using χ2 and t test respectively. Due to urinary iodine presented un-normal distribution, the urinary iodine level was expressed with median and interquartile; and then Wilcoxon test was used to assess differences of urinary iodine levels. The adjusted associations of urinary iodine level, type of salt, diet flavor, milk consumption with thyroid nodules were estimated respectively, using logistic regression model stratified by gender and resident location. Additionally, the joint associations between type of salt and milk consumption, urinary iodine and milk consumption on iodine deficiency (urine iodine concentration (UIC)<100 µg/L) were also analyzed in pooled sample and two genders. Additionally, the associations of urinary iodine level, type of salt, salt appetite, milk consumption with thyroid nodules were repeated among the subjects without diagnosed thyroid diseases, for excluding the bias from patients with diagnosed thyroid diseases. To account for the correlation of members in a same household, and the correlation of household in same district or county, we calculated robust estimates of variances with generalized estimate equation (GEE) [21].

The following variables were taken as covariates in the models of thyroid nodule risk: age, BMI, resident location, diet flavor, types of salt, dietary patterns, milk consumption. Listwise deletion was used to address the missing data in the model [22]. A value of P<0.05 was considered statistically significant. All statistical analyses were performed with SAS 9.1 (SAS Institute, Inc., Cary, NC, USA).

## Results

### The distribution of general characteristics in two groups

The table salt samples were collected from 3791 households; 3593 of those were iodized salt, and 198 were non-iodized salt. The iodine concentrations of salt range from 27.80 to 33.57 (mean = 30.09) mg/kg [23]. Finally, 74 of 3026 subjects were missing for information of thyroid nodule, thus 2974 subjects included in final analyses. The prevalence of thyroid nodule was 10.59% among children. Girls (11.89%) had higher prevalence of thyroid nodule than boys (9.26%). Subjects without thyroid nodule were younger, and participants with thyroid nodules were higher proportion from urban area (all p value <0.05); Likewise, most of subjects with thyroid nodule liked salty diet flavor, and consumed non-iodized salt (all p value <0.05); Diet patterns and milk consuming were not different between groups (Table 1).

**Table 1:** The distributions of socio-demographic characteristics among patients with thyroid nodule and no-nodule group among children.

Variables	Nodule (n = 315)	Non-nodule (n = 2659)	*P*
**Age**	12.44±2.76	11.31±3.25	<0.001
**Gender**			
male	136(43.17)	1333(50.13)	0.020
female	179(56.83)	1326(49.87)	
* missing*	0	0	
**Diet pattern^1^**			
balanced	253(80.57)	2074(78.18)	0.519
vegetarian	32(10.19)	328(12.36)	
meat	29(9.24)	251(9.46)	
* missing*	1	6	
**Resident location**			
urban area	153(448.57)	1032(38.31)	0.001
rural area	162(51.43)	1627(61.19)	
* missing*	0	0	
**Salt appetite**			
moderate	133(48.90)	1343(55.20)	0.021
salty	68(25.00)	443(18.21)	
light	71(26.10)	647(26.59)	
* missing*	43	226	
**Milk consuming**			
Yes	263(83.49)	2148(80.78)	0.246
No	52(16.51)	511(19.22)	
* missing*	0	0	
**Types of salt^2^**			
Iodized salt	294(95.15)	2569(97.24)	0.004
Non-iodized salt	21(4.85)	90(2.76)	
* missing*	0	0	

1: Vegetarian indicates that subjects consistently had vegetable diet; meat indicates that subjects consistently had meat diet; moderate indicates that subjects intermittently had vegetable diet or meat diet.

2: Iodized salt indicates that subjects consistently consumed iodized salt; Non-iodized salt indicates that subjects intermittently consumed iodized salt or consistently consumed non-iodized salt.

### The association between types of salt, milk consumption and thyroid nodule

The associations of iodized salt and milk consumption with thyroid nodule were estimated (Table 2). Non-iodized salt (OR = 1.65, 95% CI = 0.98, 2.77) was not associated with the risk of thyroid nodule among pooled samples. Consistent results were found among boys and girls, respectively. Further, no significant relationship was found between milk consumption and thyroid nodule.

**Table 2:** Adjusted associations^1^ between types of salt, salt appetite, milk consuming and thyroid nodule among boys and girls, respectively.

Variables	Nodule	Non-nodule	OR(95%CI)	*P*
	**Boys**			
**Type of salt^2^**				
Iodized salt	128(94.12)	1292(96.92)	1.00	
Non-iodized salt	8(5.88)	41(3.08)	1.47(0.64,3.40)	0.3650
**Milk consuming**				
Yes	115(84.56)	1073(80.50)	1.00	
No	21(15.44)	260(19.50)	0.92(0.55, 1.55)	0.7649
	**Girls**			
**Type of salt^2^**				
Iodized salt	166(92.74)	1277(96.30)	1.00	
Non-iodized salt	13(7.26)	49(3.70)	1.78(0.91,3.48)	0.0934
**Milk consuming**				
Yes	148(82.68)	1075(81.07)	1.00	
No	31(17.32)	251(18.93)	0.94(0.61,1.46)	0.7855
	**Pooled**			
**Type of salt^2^**				
Iodized salt	294(95.15)	2569(97.24)	1.00	
Non-iodized salt	21(4.85)	90(2.76)	1.65(0.98,2.77)	0.0598
**Milk consuming**				
Yes	263(83.49)	2148(80.78)	1.00	
No	52(16.51)	511(19.22)	0.94(0.67,1.30)	0.6896

1: Adjustment for age, sex, dietary patterns, resident location, meanwhile, the models of types of salt, milk consumption, salt appetite were adjusted each other.

2: Iodized salt indicates that subjects consistently consumed iodized salt; Non-iodized salt indicates that subjects intermittently consumed iodized salt or consistently consumed non-iodized salt.

### The association between types of salt, milk consumption and risk of thyroid nodule in different growth periods

The associations of iodized salt and milk consumption with thyroid nodule in different growth periods were estimated in Table 3. No significant association between non-iodized salt and risk of thyroid nodule was found among pooled samples in group 1 (6≤Year≤11) (OR = 1.37, 95% CI = 0.56, 3.38). Similar results were found in group 2 (12≤Year≤17) (OR = 1.96, 95% CI = 0.98, 3.89). Further, the associations were estimated in boys and girls (Table4, Table 5). No significant associations of thyroid nodule with non-iodized salt were found. Additionally, the significant relationship was not found between milk consumption and thyroid nodule.

**Table 3:** Adjusted associations^1^ between types of salt, salt appetite, milk consuming and thyroid nodule among children of different age group, respectively.

Variables	Nodule	Non-nodule	OR(95%CI)	*P*
	**6≤Year≤11**			
**Type of salt^2^**				
Iodized salt	102(93.58)	1336(96.18)	1.00	
Non-iodized salt	7(6.42)	53(3.82)	1.37(0.56,3.38)	0.4914
**Milk consuming**				
Yes	94(86.24)	1189(85.60)	1.00	
No	15(13.76)	200(14.40)	1.05(0.58,1.92)	0.8630
	**12≤Year≤17**			
**Type of salt^2^**				
Iodized salt	192(93.20)	1233(97.09)	1.00	
Non-iodized salt	14(6.80)	37(2.91)	1.96(0.98,3.89)	0.0556
**Milk consuming**				
Yes	169(82.04)	959(75.51)	1.00	
No	37(17.96)	311(24.49)	0.88(0.59,1.32)	0.5443

1: Adjustment for age, sex, dietary patterns, resident location, meanwhile, the models of types of salt, milk consumption, salt appetite were adjusted each other.

2: Iodized salt indicates that subjects consistently consumed iodized salt; Non-iodized salt indicates that subjects intermittently consumed iodized salt or consistently consumed non-iodized salt.

**Table 4:** Adjusted associations^1^ between types of salt, salt appetite, milk consuming and thyroid nodule among boys of different age group, respectively.

Variables	Nodule	Non-nodule	OR(95%CI)	*P*
	**6≤Year≤11**			
**Type of salt^2^**				
Iodized salt	45(93.75)	675(96.29)	1.00	
Non-iodized salt	3(6.25)	26(3.71)	1.34(0.35,5.18)	0.6724
**Milk consuming**				
Yes	41(85.42)	601(85.73)	1.00	
No	7(14.58)	100(14.27)	1.33(0.56,3.17)	0.5150
	**12≤Year≤17**			
**Type of salt^2^**				
Iodized salt	83(94.32)	617(97.63)	1.00	
Non-iodized salt	5(5.68)	15(2.37)	1.63(0.50,5.39)	0.4205
**Milk consuming**				
Yes	74(84.09)	472(74.68)	1.00	
No	14(15.91)	160(25.32)	0.83(0.44,1.56)	0.5608

1: Adjustment for age, dietary patterns, resident location, meanwhile, the models of types of salt, milk consumption, salt appetite were adjusted each other.

2: Iodized salt indicates that subjects consistently consumed iodized salt; Non-iodized salt indicates that subjects intermittently consumed iodized salt or consistently consumed non-iodized salt.

**Table 5:** Adjusted associations^1^ between types of salt, salt appetite, milk consuming and thyroid nodule among girls of different age group, respectively.

Variables	Nodule	Non-nodule	OR(95%CI)	*P*
	**6≤Year≤11**			
**Type of salt^2^**				
Iodized salt	57(93.44)	661(96.08)	1.00	
Non-iodized salt	4(6.56)	27(3.92)	1.34(0.39,4.59)	0.6459
**Milk consuming**				
Yes	53(86.89)	588(85.47)	1.00	
No	8(13.11)	100(14.53)	0.92(0.40,2.09)	0.8354
	**12≤Year≤17**			
**Type of salt^2^**				
Iodized salt	109(92.37)	616(96.55)	1.00	
Non-iodized salt	9(7.63)	22(3.45)	2.22(0.95,5.22)	0.0668
**Milk consuming**				
Yes	95(80.51)	487(76.33)	1.00	
No	23(19.49)	151(23.67)	0.91(0.54,1.55)	0.7337

1: Adjustment for age, dietary patterns, resident location, meanwhile, the models of types of salt, milk consumption, salt appetite were adjusted each other.

2: Iodized salt indicates that subjects consistently consumed iodized salt; Non-iodized salt indicates that subjects intermittently consumed iodized salt or consistently consumed non-iodized salt.

### Iodine status and the association between urinary iodine level and risk of thyroid nodule

The urine iodine level of subjects with thyroid nodule was not different from those without thyroid nodule. There is no difference of urinary iodine level in different groups of genders, resident location, type of salt, salt appetite and milk consumption (Table S1.). Meanwhile, 16.16% children were iodine deficiency (UIC<100 µg/L); 15.42% and 16.66% in urban and rural area, respectively. Additionally, 19.94% children were excessive iodine intake (UIC≧300 µg/L). Further, we explored the association between urinary iodine level and risk of thyroid nodule. However, after adjustment for covariates, compared with those with normal urinary iodine, the subjects with low urinary iodine level or excess iodine level were not significantly associated with thyroid nodules. Similar results were observed among boys and girls respectively (Table S2). No significant associations of thyroid nodule with UIC were observed among two age groups (Table S3, Table S4).

## Discussion

In present study, the prevalence of thyroid nodule was 10.59% among Chinese children. Girls (11.89%) had higher prevalence of thyroid nodule than boys (9.26%). Our findings indicated that subjects consumed non-iodized salt was not significant associated with the risk of thyroid nodule among Chinese children. Similar results were observed among boys and girls, respectively.

Iodized salt was still the main resource of iodine in coastal population, because the environmental iodine in study area was deficient (<10 µg/L) [24], and water iodine level in Hangzhou was in the range of 0.20–5.99 µg/L, with the median level as 2.58 µg/L [23]. Further, the average daily iodine intake from milk is 1.2 µg among 1848 subjects drinking milk; iodine content from milk accounted for 0.4% of urine iodine, and 74.4% of urine iodine came from iodized salt in adults [23]. In the present study, the associations of salt type and milk consumption with thyroid nodule were estimated among Chinese children. However, different with the findings among adults [19], significant association of non-iodized salt with thyroid nodule was not observed among Chinese children. Some prospective studies have shown that iodine supplementation does not decrease the incidence of thyroid nodules in some areas [25], this may be the result of anatomical changes made by long-standing iodine deficiency which is not expected to be resolved by iodine supplementation. Additionally, most of children under 18 years were students in schools and most diets were taken in schools. Thus, the information about non-iodized salt from questionnaires interviewed in households may be inconsistent with the actual situation. Further, the small samples of taking non-iodized salt may be another potential bias.

It has been reported that milk is one of the most significant sources used to increase iodine intake in many countries [26], [27]. However, consistent with our previous study [19], we did not observed the significant association between milk consumption and thyroid nodule. Milk may have a small effect on iodine intake in Hangzhou and that iodized salt is still the main dietary source of iodine among adults [19]. A previous study reported a mean daily iodine intake from milk of 1.2 mg, which accounted for only 0.4% of urinary iodine [23]. Another reason could be that the amount of iodine consumed in dietary supplements is not a long-term predictor of iodine intake [28].

Urinary iodine excretion is widely used as an indicator of the iodine nutritional intake. Similar with Ding Gangqiang and Wang Juanjuan [29], [30], we did not observed the difference of urinary iodine between two types of salt and patients with thyroid nodules and normal subjects. Consistent with other findings, urinary iodine was not related to the presence of thyroid nodules among our study population [31]. Our results indicated that urinary iodine status may not be associated with thyroid nodules among Chinese children. The urinary iodine excretion as determined by the iodine/creatinine ratio is influenced by additional factors (e.g., muscle mass and physical activity [32], [33]) and may be underestimated in younger and overestimated in older subjects, because creatinine excretion is negatively correlated with age. Finally, only one spot UIC might not exactly evaluate the iodine nutrition of subjects. The accurate correlations of thyroid nodules with non-iodized salt among Chinese children need more deep and large samples studies.

## Limitations

There are some limitations in this study. The number and mass size of thyroid nodule were not recorded in the investigation. The associations between iodized salt and number and mass size of thyroid nodule were not analyzed, which might reveal the extent of influence of the iodized salt on thyroid nodule. In addition, the classification of thyroid nodules was not included, and it was no way to find the different associations between iodized salt and different kinds of thyroid nodules. Additionally, other thyroid diseases were not included in present analyzed. It would be distorted the association between iodized salt and thyroid diseases.

## Conclusions

No statistical association between non-iodized salt and thyroid nodules was observed among Chinese children. There is no significant relationships between milk consumption, urinary iodine status and thyroid nodules among Chinese children.

## Supporting Information

Table S1 **The comparison of urine iodine levels among the children and adolescent.**
(DOCX)

Table S2 **Adjusted associations between urinary iodine level and thyroid nodule among boys and girls.**
(DOCX)

Table S3 **Adjusted associations between urinary iodine level and thyroid nodule among different develop stage of girls.**
(DOCX)

Table S4 **Adjusted associations between urinary iodine level and thyroid nodule among different develop stage of boys.**
(DOCX)
