# Supplementary material for: The Association of Thyroid Nodule with Non-Iodized Salt among Chinese Children
Source: PLoS One. 2014 Jul 28;9(7):e102726. doi: 10.1371/journal.pone.0102726 (PMC4113344; doi:10.1371/journal.pone.0102726)
Supplement: Table S1 — The comparison of urine iodine levels among the children and adolescent. (DOCX) [file pone.0102726.s001.docx]

| Table S1.The comparison of urine iodine levels among the children and adolescent. | | |
| --- | --- | --- |
| **Variables** | **Urinary iodine level**  **median(interquartile)** | ***p* value ^1^** |
| **Thyroid Nodule** |  |  |
| Yes | 189(129,280) | 0.7235 |
| No | 188(122,274) |  |
| **Gender** |  |  |
| male | 190(127,267) | 0.5279 |
| female | 182(117,281) |  |
| **Resident location** |  |  |
| urban area | 182(121,267) | 0.7717 |
| rural area | 189(124,276) |  |
| **Types of salt^2^** |  |  |
| Iodized salt | 188(124,274) | 0.0551 |
| Non-iodized salt | 146.5(94,247) |  |
| **Salt appetite** |  |  |
| moderate | 188(125,274) | 0.5214 |
| salty | 194(125,275) |  |
| light | 179(117,271) |  |
| **Milk consumption** |  |  |
| Yes | 186(121,271) | 0.0858 |
| No | 190(126,284) |  |
| ^1:^ Wilcoxon test was used for the value;  ^2:^ Iodized salt indicates that subjects consistently consumed iodized salt; Non-iodized salt indicates that subjects intermittently consumed iodized salt or consistently consumed non-iodized salt. | | |
