# Supplementary material for: The Association of Thyroid Nodule with Non-Iodized Salt among Chinese Children
Source: PLoS One. 2014 Jul 28;9(7):e102726. doi: 10.1371/journal.pone.0102726 (PMC4113344; doi:10.1371/journal.pone.0102726)
Supplement: Table S2 — Adjusted associations between urinary iodine level and thyroid nodule among boys and girls. (DOCX) [file pone.0102726.s002.docx]

| Table S2. Adjusted associations^1^ between urinary iodine level and thyroid nodule among boys and girls. | | | | |
| --- | --- | --- | --- | --- |
| **Urinary iodine^2^ (μg/L)** | **Nodule** | **Non-nodule** | **OR(95%CL)** | ***P*** |
|  | **Boys** | | | |
| Normal | 44(33.59) | 491(37.65) | 1.00 |  |
| Low | 18(13.74) | 196(15.03) | 0.95(0.50,1.82) | 0.8819 |
| High | 42(32.06) | 389(29.83) | 1.13(0.67,1.89) | 0.6535 |
| Excess | 27(20.61) | 228(17.48) | 1.32(0.76,2.30) | 0.3217 |
|  |  |  |  |  |
|  | **Girls** | | | |
| Normal | 70(40.94) | 490(37.78) | 1.00 |  |
| Low | 28(16.37) | 227(17.50) | 0.82(0.49,1.38) | 0.4503 |
| High | 34(19.88) | 295(22.74) | 0.80(0.49,1.29) | 0.3573 |
| Excess | 39(22.81) | 285(21.97) | 0.96(0.59,1.57) | 0.8825 |
|  |  |  |  |  |
|  | **Pooled** | | | |
| Normal | 114(37.75) | 981(37.72) | 1.00 |  |
| Low | 46(15.23) | 423(16.26) | 0.88(0.59,1.31) | 0.5236 |
| High | 76(25.17) | 684(26.30) | 0.94(0.66,1.32) | 0.7104 |
| Excess | 66(21.85) | 513(19.72) | 1.09(0.76,1.57) | 0.6468 |
| ^1:^ Adjusted for age, BMI, resident location, salt appetite, types of salt, dietary patterns, milk consuming  ^2:^ urinary iodine levels: low: <100μg/L, normal: 100~ μg/L, high: 200~ μg/L and excess: ≥300μg/L. | | | | |
