# Supplementary material for: The Association of Thyroid Nodule with Non-Iodized Salt among Chinese Children
Source: PLoS One. 2014 Jul 28;9(7):e102726. doi: 10.1371/journal.pone.0102726 (PMC4113344; doi:10.1371/journal.pone.0102726)
Supplement: Table S3 — Adjusted associations between urinary iodine level and thyroid nodule among different develop stage of girls. (DOCX) [file pone.0102726.s003.docx]

| Table S3.Adjusted associations^1^ between urinary iodine level and thyroid nodule among different develop stage of girls. | | | | |
| --- | --- | --- | --- | --- |
| **Urinary iodine^2^ (μg/L)** | **Nodule** | **Non-nodule** | **OR(95%CL)** | ***P*** |
|  | **6≤Year≤11** | | | |
| Normal | 18(31.58) | 265(39.32) | 1.00 |  |
| Low | 11(19.30) | 125(18.55) | 1.46(0.60,3.56) | 0.4099 |
| High | 13(22.81) | 142(21.07) | 1.58(0.67,3.73) | 0.2940 |
| Excess | 15(26.30) | 142(21.07) | 1.63(0.69,3.82) | 0.2615 |
|  |  |  |  |  |
|  | **12≤Year≤17** | | | |
| Normal | 52(45.61) | 225(36.12) | 1.00 |  |
| Low | 17(14.91) | 102(16.37) | 0.79(0.41,1.51) | 0.4676 |
| High | 21(18.42) | 153(24.56) | 0.66(0.35,1.25) | 0.1990 |
| Excess | 24(21.05) | 143(22.95) | 0.84(0.45,1.56) | 0.5780 |
| ^1:^ Adjusted for age, BMI, resident location, salt appetite, types of salt, dietary patterns, milk consuming  ^2:^ urinary iodine levels: low: <100μg/L, normal: 100~ μg/L, high: 200~ μg/L and excess: ≥300μg/L. | | | | |
