# Supplementary material for: The Association of Thyroid Nodule with Non-Iodized Salt among Chinese Children
Source: PLoS One. 2014 Jul 28;9(7):e102726. doi: 10.1371/journal.pone.0102726 (PMC4113344; doi:10.1371/journal.pone.0102726)
Supplement: Table S4 — Adjusted associations between urinary iodine level and thyroid nodule among different develop stage of boys. (DOCX) [file pone.0102726.s004.docx]

| Table S4.Adjusted associations^1^ between urinary iodine level and thyroid nodule among different develop stage of boys. | | | | |
| --- | --- | --- | --- | --- |
| **Urinary iodine^2^ (μg/L)** | **Nodule** | **Non-nodule** | **OR(95%CL)** | ***P*** |
|  | **6≤Year≤11** | | | |
| Normal | 15(34.09) | 263(38.12) | 1.00 |  |
| Low | 8(18.18) | 100(14.49) | 1.28(0.50,3.31) | 0.6070 |
| High | 16(36.36) | 210(30.43) | 1.23(0.55,2.74) | 0.6114 |
| Excess | 5(11.36) | 117(16.96) | 0.81(0.28,2.32) | 0.6970 |
|  |  |  |  |  |
|  | **12≤Year≤17** | | | |
| Normal | 29(33.33) | 228(37.13) | 1.00 |  |
| Low | 10(11.49) | 96(15.64) | 0.68(0.27,1.70) | 0.4090 |
| High | 26(29.89) | 179(29.15) | 1.07(0.54,2.09) | 0.8503 |
| Excess | 22(25.29) | 111(18.08) | 1.72(0.86,3.42) | 0.1225 |
| ^1:^ Adjusted for age, BMI, resident location, salt appetite, types of salt, dietary patterns, milk consuming  ^2:^ urinary iodine levels: low: <100μg/L, normal: 100~ μg/L, high: 200~ μg/L and excess: ≥300μg/L. | | | | |
